# Streamlined downstream process for efficient and sustainable (Fab')_2_ antivenom preparation

**DOI:** 10.1590/1678-9199-jvatitd-2020-0025

**Published:** 2020-07-27

**Authors:** Tihana Kurtović, Marija Brgles, Maja Lang Balija, Stephanie Steinberger, Dora Sviben, Martina Marchetti-Deschmann, Beata Halassy

**Affiliations:** 1 Center for Research and Knowledge Transfer in Biotechnology, University of Zagreb, Zagreb, Croatia.; 2 Faculty of Technical Chemistry, Institute of Chemical Technologies and Analytics, TU Wien, Vienna, Austria.

**Keywords:** Antivenom downstream processing, F(ab')_2_ immunotherapy, Hyperimmune plasma, Ion-exchange chromatography, Mass spectrometry

## Abstract

**Background:**

Antivenoms are the only validated treatment against snakebite envenoming. Numerous drawbacks pertaining to their availability, safety and efficacy are becoming increasingly evident due to low sustainability of current productions. Technological innovation of procedures generating therapeutics of higher purity and better physicochemical characteristics at acceptable cost is necessary. The objective was to develop at laboratory scale a compact, feasible and economically viable platform for preparation of equine F(ab')_2_ antivenom against *Vipera ammodytes ammodytes* venom and to support it with efficiency data, to enable estimation of the process cost-effectiveness.

**Methods:**

The principle of simultaneous caprylic acid precipitation and pepsin digestion has been implemented into plasma downstream processing. Balance between incomplete IgG breakdown, F(ab')_2_ over-digestion and loss of the active drug's protective efficacy was achieved by adjusting pepsin to a 1:30 substrate ratio (*w*/*w*) and setting pH at 3.2. Precipitation and digestion co-performance required 2 h-long incubation at 21 °C. Final polishing was accomplished by a combination of diafiltration and flow-through chromatography. *In vivo* neutralization potency of the F(ab')_2_ product against the venom's lethal toxicity was determined.

**Results:**

Only three consecutive steps, performed under finely tuned conditions, were sufficient for preservation of the highest process recovery with the overall yield of 74%, comparing favorably to others. At the same time, regulatory requirements were met. Final product was aggregate- and pepsin-free. Its composition profile was analyzed by mass spectrometry as a quality control check. Impurities, present in minor traces, were identified mostly as IgG/IgM fragments, contributing to active drug. Specific activity of the F(ab')_2_ preparation with respect to the plasma was increased 3.9-fold.

**Conclusion:**

A highly streamlined mode for production of equine F(ab')_2_ antivenom was engineered. In addition to preservation of the highest process yield and fulfillment of the regulatory demands, performance simplicity and rapidity in the laboratory setting were demonstrated. Suitability for large-scale manufacturing appears promising.

## Background

Envenoming following snakebite is a very common medical emergency responsible for enormous suffering all over the world. In 2017 the WHO added this public health problem to its list of Category A neglected tropical diseases [1]. In 2019 a comprehensive strategy was developed to reduce the number of snakebite deaths and disability cases by 50% before 2030.

Passive immunotherapy with animal-derived antivenoms, containing either immunoglobulin G (IgG) or its derivative fractionation products, F(ab')_2_ or Fab, has been the only validated treatment for snakebite envenoming for decades [2]. Antivenoms were recognized as essential medicines more than a century ago and number of technological platforms for their production are available. Yet, we are currently faced with severe shortages, especially in sub-Saharan Africa and parts of Asia, but even in high-income countries. Two moments are critical. There is a need for implementation of better immunization protocols yielding plasma material of higher potency. Advances in the design of sustainable manufacturing strategies generating available, safe and efficient life-saving medications are another necessity. One of the most widely used approaches for antivenom production as the initial step employs a salting-out procedure [3-7], which is associated with a low-purity profile of the final product or excessive formation of aggregates [8, 9]. Caprylic acid as an alternative precipitating agent [10] has been introduced into the preparation of a whole series of equine or ovine IgG- or F(ab')_2_-based antivenoms [11-17]. Purification principles based on caprylic acid have been proven more beneficial than salt-mediated methods due to selective precipitation of non-IgG plasma proteins [18, 19]. Thus, the active drug (IgG) remains in a solubilized state, preserving conformation and/or structural stability. 

Some antivenom manufacturers perform enzyme-mediated removal of the Fc portion from IgG molecules that can be accomplished upon their purification [20], on unfractionated plasma [16, 20, 21] or simultaneously with the precipitation of unwanted proteins using caprylic acid, as demonstrated on ovine serum [17]. Aside from the doubtful role of the Fc fraction in adverse reactions [18, 19, 22, 23], its removal reduces the quantity of foreign material in the product intended for use in humans, thus increasing specific activity and at least partially contributing to safety improvement. Although both F(ab')_2_ and Fab antivenoms are currently involved in snakebite management [20], fewer laboratories have adopted the production of the latter ones for commercial purposes [24]. Specifically, in vast majority of cases F(ab')_2_ antivenoms have been considered more efficacious in a clinical setting owing to their pharmacokinetic properties, which are valid for longer retention in systemic circulation, and possession of two antigen-binding sites, mediating the venom-neutralization activity through formation of large, stable and precipitable complexes [20, 25].

Additional procedures, most notably ion-exchange chromatography, have been introduced to increase the product's purity after an initial processing step, such as salting-out or caprylic acid fractionation of pepsin-digested plasma [16, 24]. The anion-exchange approach is favored for pragmatic reasons, since contaminants bind to the column, whereas IgGs or their fragments, present at much higher concentrations, remain unhindered [24, 26]. Affinity chromatography for the purification of F(ab')_2_ fragments has also been suggested [27], although its application for industrial manufacture is largely impractical from the standpoint of cost [24].

Recently, we have proposed a refinement strategy [28] as an attempt to offer a new perspective for overcoming cost-efficiency difficulties associated with antivenom production. It has been demonstrated on equine plasma and consisted of caprylic acid-mediated fractionation, depletion of precipitating agent from the IgG-enriched fraction, pepsin digestion, diafiltration of the F(ab')_2_-based preparation and its final refinement by the flow-through chromatography. Although all regulatory requirements concerning yield, purity and aggregate content have been successfully fulfilled, we were challenged to lay the foundations for the development of an even more sustainable approach. The main intention was to achieve it through simplification and reduction of processing steps, performed under cost-reducing and finely tuned conditions. To the best of our knowledge, for the first time, caprylic acid precipitation has been undertaken simultaneously with pepsin digestion and applied to manufacturing of equine antivenom. Following diafiltration and chromatography, F(ab')_2_-based final product of high yield and quality was obtained. Furthermore, the recovery of active drug was precisely quantified in each processing step, enabling accurate estimation of the procedure's cost-effectiveness.

## Methods

### Snake venom, plasma pools, animals and reagents

*Vipera ammodytes ammodytes* venom samples, two pools of *V. ammodytes ammodytes*-specific hyperimmune horse plasma (HHP) and NIH Ola/Hsd mice (18-20 g) of both sexes for lethal toxicity neutralization assay were obtained from the Institute of Immunology Inc. (Croatia). Bovine serum albumin (BSA), caprylic acid (≥ 98%), dithiothreitol (DTT), iodoacetamide (IAA), 2-(N-morpholino) ethanesulphonic acid (MES) monohydrate, *o*-phenylenediamine dihydrochloride (OPD), thimerosal, Tris base and Tween 20 were from Sigma-Aldrich (USA). Pepsin (from porcine gastric mucosa, 0.7 Ph. Eur. U mg^-1^) was from Merck (Germany). Goat anti-horse F(ab')_2_ IgG conjugated with horseradish peroxidase (HRP) was from *antibodies-online* (Germany). All other chemicals for buffers and solutions were from Kemika (Croatia), unless otherwise stated.

### Optimization of F(ab')_2_ preparation by caprylic acid precipitation and pepsin digestion co-performance

As a starting point, incubation duration (from 1 to 6 h or overnight) and temperature (23 or 37 °C) were investigated. HHP was heated at 56 °C for 1 h. After centrifugation at 3,200 × *g* for 40 min and discarding the pellet, supernatant was acidified to pH 3.2 using 6 M HCl. Subsequently, caprylic acid and pepsin in 0.15 M NaCl were added while vigorously stirring (750 rpm) in a thermomixer (Eppendorf, Germany). In every 2-fold diluted reaction mixture (*V* = 1 mL), the final concentration of caprylic acid was 2% (*V*/*V*) and the pepsin-to-IgG ratio was 1:75 (*w*/*w*). At timed intervals, solutions were neutralized by mixing with 1 M Tris base and centrifuged again (2,800 × *g*, 45 min). Supernatants were collected and filtered through cellulose acetate filters with a pore size of 5 μm (Sartorius, Germany). 

Subsequently, the pepsin-to-IgG ratio (X1) and temperature (X2), each at two levels (marked with minus (-) for the lower and plus (+) for the higher level), were further selected to study their impact on the outcome of a simultaneously performed precipitation and digestion. The incubation duration producing the highest yield (*i.e.* 2 h) was selected according to results from the experiment described above. Investigated factors' values were 1:30 or 1:75 (*w*/*w*) for the pepsin-to-IgG ratio and 21 or 25 °C for temperature. A full factorial design was employed [29], resulting in 4 experimental runs. Each was performed in triplicate. The main effect of each factor was calculated according to Eq. (1),

$$E_{X}\;=\;\frac{2*\sum {\bar{Y}}_{j}^{+}}{n}-\;\frac{2*\sum {\bar{Y}}_{j}^{-}}{n}$$ (1)

where index *X* represents factors 1 or 2, *n* is the total number of experimental runs (4), while ${\bar{Y}}_{j}^{-}$ and ${\bar{Y}}_{j}^{+}$ are F(ab')_2_ yields (%) obtained at the - and + level of each factor. The significance of the given factors was determined by means of ANOVA using the software Statistica 13.5 (StatSoft, TIBCO Software Inc.). 

Protein products of simultaneous precipitation and digestion step were analyzed by SDS-PAGE. Preliminarily, in low-scale experiments, yield and purity were monitored by size-exclusion chromatography (SEC). When optimal conditions were achieved, the procedure was scaled up 20-fold and F(ab')_2_ quantity was measured by ELISA (as described in "ELISA assay for F(ab')_2_ content determination" section). 

### Diafiltration and flow-through chromatography for the final polishing

Following simultaneous precipitation and digestion of HHP, the supernatant (crude F(ab')_2_) was diafiltrated into 20 mM MES + 0.15 M NaCl, pH 5.0, using a Vivaspin centrifugal concentrator (Sartorius, Germany) with a molecular weight cutoff (MWCO) polyethersulphone membrane of 50 kDa. The resultant preparation was marked as pure F(ab')_2_. 

Diafiltrated samples of pure F(ab')_2_ were loaded (2 mL per run) to pre-equilibrated CIM QA disk (*V* = 0.34 mL; BIA Separations, Slovenia) with 20 mM MES + 0.15 M NaCl binding buffer, pH 5.0, at a flow rate of 2 mL min^-1^ on an ÄKTA chromatography system (GE Healthcare, USA). The absorbance was monitored at 280 nm. After collecting the flow-through fractions (referred to as ultrapure F(ab')_2_), the bound components were eluted from the column with binding buffer containing 1 M NaCl. 

### Electrophoretic and chromatographic profiling

The purity of the F(ab')_2_ sample (20 μg) in each processing step was examined by SDS-PAGE analysis according to the manufacturer's protocol using MES-containing running buffer in combination with 4-12% Bis-Tris gel under non-reducing conditions in an Xcell SureLock Mini-Cell (Invitrogen, USA). Staining was carried out with acidic Coomassie Brilliant Blue (CBB) R250 or, alternatively, AgNO_3_ for "negative" detection of pepsin remains [30]. As the first dimension of 2D gel electrophoresis, a ZOOM IPGRunner Mini-Cell (Invitrogen, USA) was used in combination with immobilized pH gradient (IPG) strip (7 cm long, linear pH 3-10; Invitrogen, USA) rehydrated with F(ab')_2_ sample (350 μg). The following step voltage protocol was applied: 200 V for 20 min, 450 V for 15 min, 750 V for 15 min and 2,000 V for 6 h. For the second dimension, 4-12% Bis-Tris gel was used as described above after reduction (20 mM DTT) and alkylation (125 mM IAA). CBB R250-stained protein spots served as starting material for mass spectrometry (MS) analysis.

SEC analysis, which was employed for monitoring of F(ab')_2_ purity in all three purification steps, was performed on TSK-Gel G3000SWXL column (7.8 × 300 mm; Tosoh Bioscience, Japan) with 0.1 M phosphate-sulfate running buffer, pH 6.6, at a flow rate of 0.5 mL min^-1^ on a Waters HPLC system (Waters, USA). The sample (2 mg mL^-1^) was loaded to column in a volume of 50 μL per run. The effluent was monitored at 280 nm. For determination of F(ab')_2_ molecular weight, thyroglobulin (*M*_r_ 665,000), γ-globulin (*M*_r_ 150,000), ovalbumin (*M*_r_ 44,300) and ribonuclease A (*M*_r_ 13,700) were used as standards. 

### MALDI-MS analysis

Excised protein spots obtained by 2D gel electrophoresis of F(ab')_2_ sample were prepared and analyzed by MALDI MS/MS on an ultrafleXtreme (Bruker, Germany), as described by Kurtović et al. [28]. Proteins were considered to be confidently identified after submitting peptide sequencing data to Mascot (taxonomy "other mammalians") and receiving statistically significant scores for at least two peptides per protein. If data on only one peptide were available, theoretical molecular masses and pI values of the database entry were compared to experimental 2D gel electrophoresis results and, in the case of agreement, the identification was considered to be correct. 

### Protein, IgG and F(ab')_2_ concentration determination

Throughout the isolation procedure, the total protein concentration was estimated spectrophotometrically by use of the Eq. (2) [31],

$$\gamma \;[mg\;{mL}^{-1}mL]\;=\;(A_{228.5\;nm}\;–A_{234.5\;nm})\;\times \;f\;\times \;dilution\;factor$$ (2)

where Ehresmann's factor "*f*" for equine IgG of 0.2553 was used [32].

The IgG concentration in each HHP was measured by ELISA, as described previously [28].

F(ab')_2_ content in samples from HHP processing was determined by ELISA according to the protocol from Kurtović et al. [28]. European viper venom antiserum (Zagreb antivenom; Institute of Immunology Inc., Croatia) was used as the standard. Considering composition differences in subclass and/or venom-specific antibody distribution between each investigated sample and the standard, a recently developed principle for F(ab')_2_ estimation that uses sample-specific correction was applied [32]. Namely, F(ab')_2_ preparation of the highest purity (referred to as ultrapure F(ab')_2_), which was processed from the respective HHP and precisely quantified, served as the internal reference. Its concentration was calculated as: [SEC-determined purity in percentage / 100%) × *γ*(protein) ].

Yield was calculated as: [ (*γ*(F(ab')_2_) × dilution factor) / (*γ*(IgG) in HHP × 0.67) ] × 100%. IgG and F(ab')_2_ concentrations were measured by the respective ELISA assays, as described above. Purities of intermediates and the final product were expressed as: [*γ*(F(ab')_2_) / *γ*(protein) ] × 100%, where total protein concentration was determined spectrophotometrically according to Eq. (2). SEC monitoring for purity profiling was also included.

### ED_50_ test

The potential of HHP and final F(ab')_2_ preparation to neutralize the venom's lethal toxicity was determined by the lethal toxicity neutralization assay in mice [33]. The lethal toxicity neutralization potency (*R*) was expressed as the number of LD_50_ venom doses that can be neutralized by 1 mL of undiluted sample and calculated by Eq. (3),

$$R=(Tv-1)\;/\;{ED}_{50}$$ (3)

where Tv represents the number of LD_50_ venom doses inoculated per mouse [34]. The *R*-value was used as a measure of the protective efficacy of each sample. Specific activity (LD_50_ mg^-1^) was calculated as a ratio of *R*-value and either active drug (F(ab')_2_) or total protein concentration.

## Results

### Optimization of F(ab')_2_ preparation

**Figure 1.**
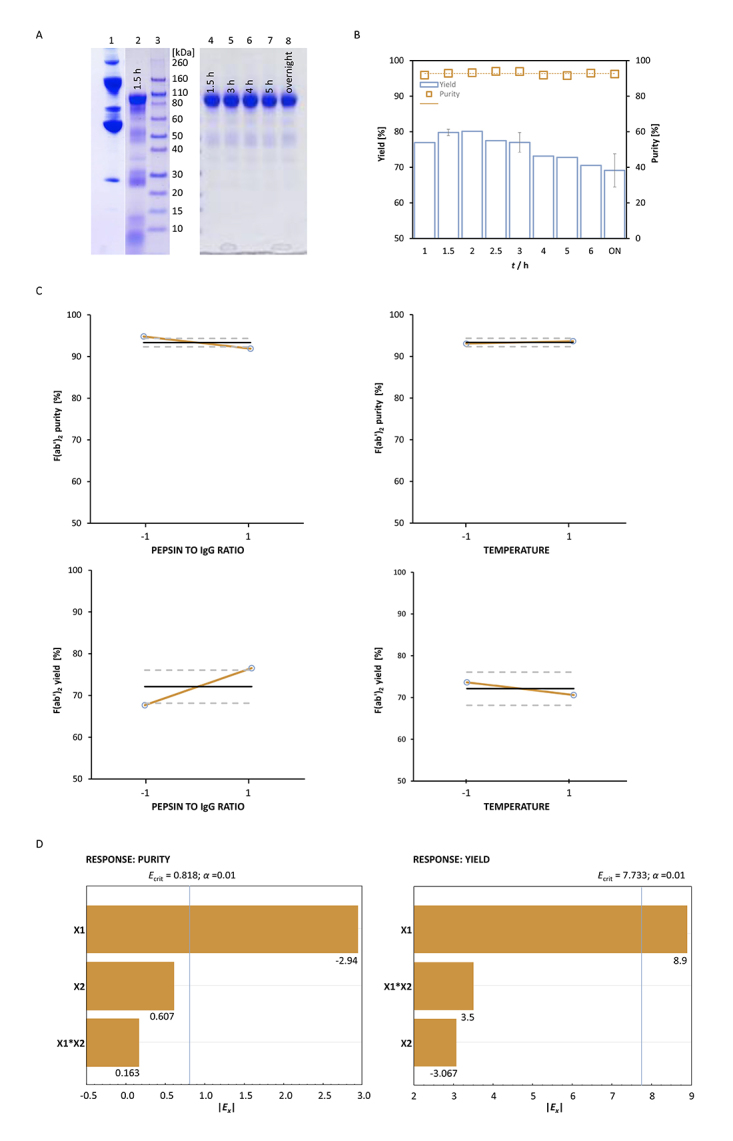
Optimization of F(ab')_2_ preparation**. (A)** SDS-PAGE analysis of samples (20 μg) obtained by simultaneous caprylic acid precipitation and pepsin digestion of HHP at 37 or 23 °C. Lane 1, HHP; Lane 2, sample obtained at 37 °C with 1.5 h-long incubation; Lane 3, molecular weight standards; lanes 4-8, samples obtained at 23 °C with duration of incubation as indicated. Staining was performed with CBB R250. **(B)** Time-course of SEC-determined yields and purities of F(ab')_2_ preparations obtained by precipitation and digestion co-performance at 23 °C following diafiltration on a 50 kDa membrane. Results are given as mean +/- standard error. **(C)** Optimization of precipitation and digestion co-performance step with respect to the pepsin-to-IgG ratio and temperature studied according to full factorial experimental design. Mean yield (ο) of active drug obtained post-diafiltrationally at higher and lower level of each experimental factor (X1 - pepsin-to-IgG ratio, X2 - temperature) in comparison to mean value (full line) and 95% confidence interval (dashed lines) from the overall set of experiments. **(D)** Pareto plot of effect estimates. The absolute value of the main effect for each factor (|*E*_*x*_ |) is presented in horizontal columns. The critical effect values for significance level of *α* = 0.01 is marked by a vertical line.

Initially, all experimental conditions were adopted from our previously developed downstream processing strategy in which extraction of IgGs and their subsequent enzymatic cleavage were performed as two separate operating units with an interposed diafiltration-mediated removal of precipitating agent [28]. Namely, heat-treated HHP, acidified to pH 3.2, was fractionated and digested at once by simultaneous incubation with 2% caprylic acid (*V*/*V*) and pepsin, the latter in a quantity 75-times lower than the measured IgG content (1:75, *w*/*w*). Temperature was set to 37 °C. SDS-PAGE analysis of samples from 1.5 and 2 h incubation durations revealed major contaminations of F(ab')_2_ products (Fig. 1A, left panel). In addition, following diafiltration on a 50 kDa membrane, total protein concentration in each retentate was decreased by approximately 60%, indicating loss of the target component.

In the neasxt experiment, the impact of temperature reduction on the efficiency of contaminants removal and boosting of F(ab')_2_ yield was examined. Incubation was performed at 23 °C from 1 to 6 h or overnight. The other operating parameters remained unchanged. SDS-PAGE analysis revealed that even a short reaction time was sufficient to achieve precipitation of almost all unwanted plasma proteins and complete hydrolysis of IgG material remaining in supernatant (Fig. 1A, right panel). High purity of around 90% was achieved post-diafiltrationally, while yield declined with prolongation of incubation (Fig. 1B). The highest one of around 80% was obtained when digestion was terminated after 2 h. 

A previous experiment indicated appropriateness of 2-h long incubation at 23 °C. Finally, the pepsin-to-IgG ratio (1:30 or 1:75, *w*/*w*) and temperature (21 or 25 °C) were examined according to the full factorial design. Their impact on F(ab')_2_ yield is presented in Figure 1C. The main effect estimates *E*_*X*_ for each factor are indicated in Figure 1D. Statistical analysis showed that only factor X1 (pepsin-to-IgG ratio) had significant influence on response variables (*α* = 0.01) in the tested range of its values. A higher pepsin concentration improved yield. At the same time, it slightly impaired purity, probably due to the contribution of the enzyme itself. Accordingly, a pepsin-to-IgG ratio of 1:30 (*w*/*w*) and temperature of 21 °C, which positively affected yield, although not significantly, were selected as optimal conditions.

### F(ab')_2_ preparation under optimized conditions and final polishing

**Figure 2.**
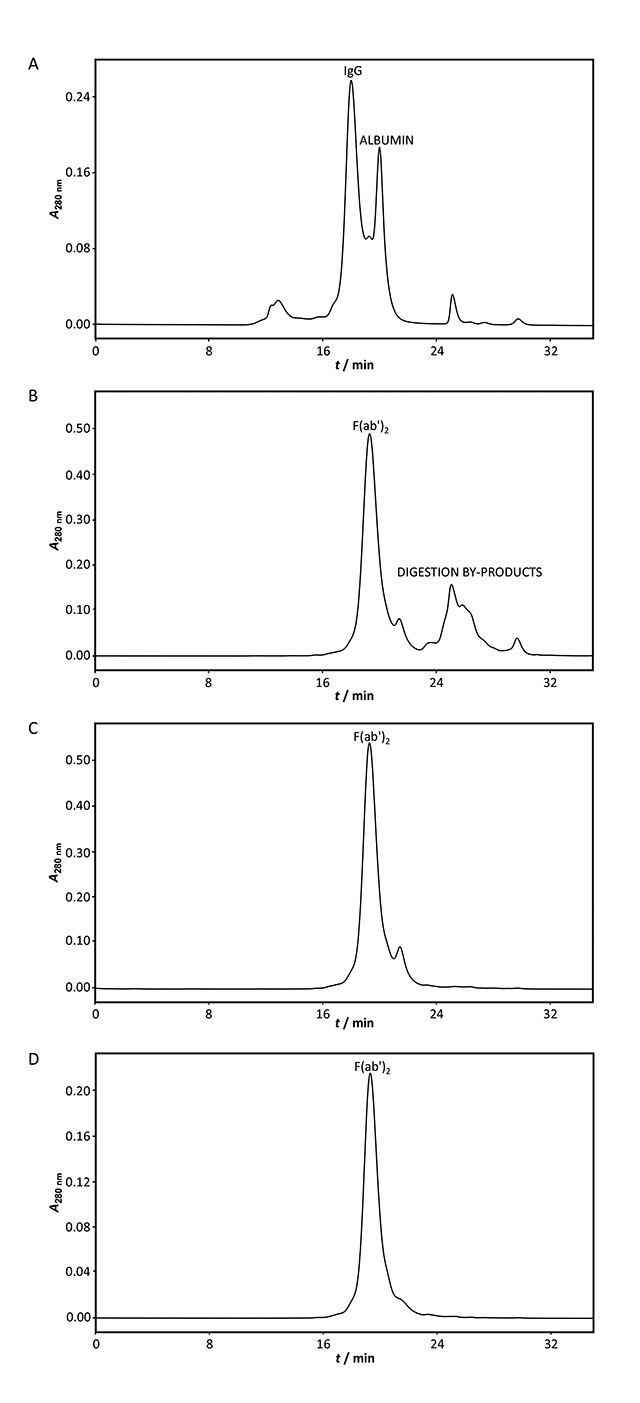
The assessment of purification steps by size-exclusion chromatography. The analysis was performed on a TSK-Gel G3000SWXL column (7.8 × 300 mm) with 0.1 M phosphate-sulfate running buffer, pH 6.6, at a flow rate of 0.5 mL min^-1^. The sample (2 mg mL^-1^) was loaded to the column in a volume of 50 μL per run. **(A)** Heat-treated plasma. **(B)** F(ab')_2_ fraction obtained by simultaneous 2% caprylic acid (*V*/*V*) precipitation and pepsin digestion before (crude IgG) and **(C)** after diafiltration using a 50 kDa membrane (pure IgG). **(D)** Ultrapure F(ab')_2_ preparation - flow-through fraction from anion-exchange chromatography performed at pH 5.0. Detection: UV at 280 nm.

**Figure 3.**
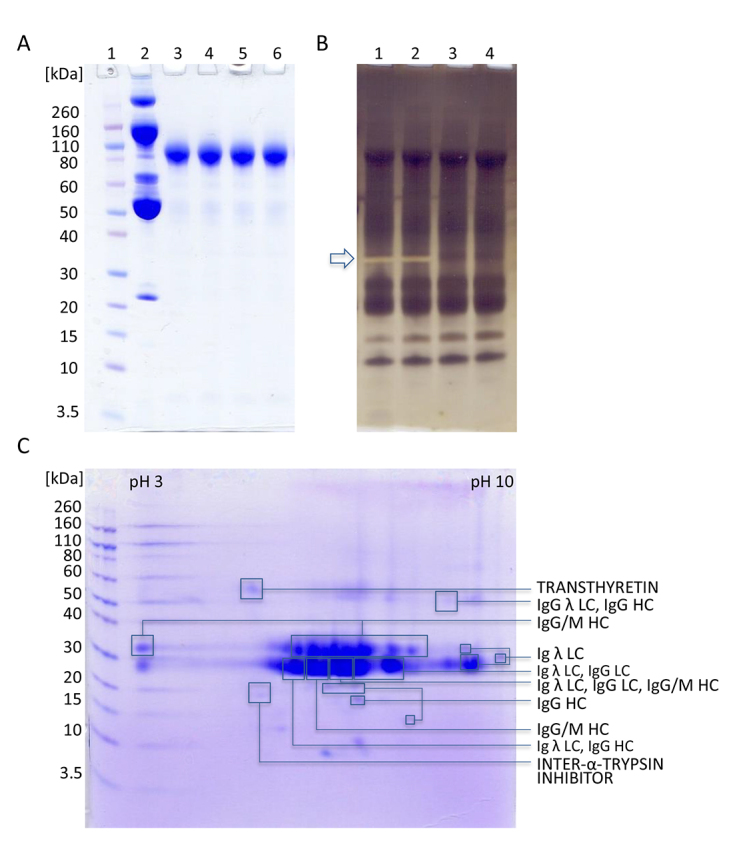
Gel electrophoresis of representative samples from purification process on 4-12% gel. **(A)** SDS-PAGE analysis of HHP (40 μg) and F(ab')_2_ fractions (20 μg) under non-reducing conditions with CBB R250 staining. Lane 1, molecular weight standards; Lane 2, HHP; Lane 3, F(ab')_2_ fraction obtained by simultaneous 2% caprylic acid (*V*/*V*) precipitation and pepsin digestion (crude F(ab')_2_); Lane 4, F(ab')_2_ fraction after diafiltration (pure F(ab')_2_); lanes 5 and 6, F(ab')_2_ preparation using CIM QA chromatography (ultrapure F(ab')_2_). **(B)** SDS-PAGE analysis of F(ab')_2_ fractions (20 μg) under non-reducing conditions with silver staining. Lanes 1-4 correspond to lanes 3-6 from (A). "Negatively" silver-stained bands corresponding to pepsin are denoted by arrow. **(C)** 2D gel electrophoresis of the final product. In the first dimension F(ab')_2_ (350 μg) was focused using IPG strip under denaturing conditions (linear pH 3-10). Prior second dimension IPG strip was reduced, alkylated and loaded to a 4-12% gel. Proteins were detected with CBB R250 and identified by MS/MS analysis (LC = light chain, HC = heavy chain). Molecular mass markers are on the left side.

In the first step, fractionation of HHP (Fig. 2A) with 2% (*V*/*V*) caprylic acid and digestion of plasma proteins, utilizing pepsin at a concentration 30-times lower than the measured IgG content (1:30, *w*/*w*), were co-performed. The established conditions, pH of 3.2 in the reaction mixture and 21 °C, proved supportive for concerted precipitation and enzymatic cleavage. For a 2 h incubation duration, a significant reduction of contamination in the supernatant occurred, while the whole IgG fraction that remained in solution underwent limited hydrolysis, resulting in F(ab')_2_ fragments as the dominant product (crude F(ab')_2_) (Figs. 2B and 3A) at the expected molecular weight (91.7 ± 1.7 kDa, *n* = 12) and with satisfactory recovery (Table 1). Specifically, the average yield of the first step was around 85%. 

Enhancement of purity approaching 90% was achieved through diafiltration-mediated removal of small molecular fragments of Fc and digestion by-products of albumin and other plasma proteins (Figs. 2C and 3A, Table 1). Following diafiltration as an intermediate step, pure F(ab')_2_ was obtained. SEC revealed absence of aggregates from F(ab')_2_-enriched product, either crude (Fig. 2B) or pure (Fig. 2C).

In the third purification step residual by-product contaminants, especially pepsin, were removed from the pure F(ab')_2_ by anion-exchange chromatography. Optimal conditions were adopted from our previous research, *i.e.*, exclusive adsorption of the enzyme and other acidic impurities to the column while the target compound passes unhindered [28]. Final polishing produced an active drug (ultrapure F(ab')_2_) of approximately 97% purity and without any loss (Fig. 2D, Table 1). The recovery of CIM QA chromatography step was 100.9 ± 4.3% (*n* = 11). The overall yield was 74% (Table 1). The final product was free from aggregates (Fig. 2D) and depleted from pepsin, as confirmed by SDS-PAGE analysis and absence of ''negative'' band at position corresponding to its molecular weight following silver staining (Fig. 3B). 

**Table 1.** Purities and yields of the intermediates and the final product obtained by developed downstream processing protocol. Two different plasma pools (HHP1 and HHP2) were used. IgG purities in HHPs, before and after thermal treatment, are expressed as means from *n* measurements +/- 95% confidence interval. IgG yields in thermally treated HHPs, as well as F(ab')_2_ purities and yields, are given as means from *n* independent process performances +/- 95% confidence interval.

Processing step	Product	IgG/F(ab')_2_ purity (%)	IgG/F(ab')_2_ yield (%)*	Overall IgG/F(ab')_2_ yield (%)
	HHP1	47.8 ± 2.9 (*n* = 17)	n.a.	n.a.
	HHP2	41.5 ± 2.8 (*n* = 15)	n.a.	n.a.
Heat denaturation	Thermally treated HHP1	46.9 ± 4.1 (*n* = 9)	91.6 ± 2.4 (*n* = 8)	91.6 ± 2.4 (*n* = 8)
	Thermally treated HHP2	44.0 ± 3.2 (*n* = 26)		
Precipitation and digestion	Crude F(ab')_2_	60.4 ± 1.7 (*n* = 5)	85.2 ± 6.1 (*n* = 6)	76.8 ± 5.5 (*n* = 6)
Diafiltration	Pure F(ab')_2_	86.7 ± 6.4 (*n* = 6)	95.9 ± 6.1 (*n* = 6)	73.6 ± 6.1 (*n* = 6)
Anion-exchange HPLC	Ultrapure F(ab')_2_	97.3 ± 4.0 (*n* = 8)	100.9 ± 4.3 (*n* = 11)	74.3 ± 5.9 (*n* = 8)

*Yield in relation to the preceding processing step

### MS/MS analysis of the final product

MS/MS analysis of protein spots obtained by 2D gel electrophoresis, intentionally performed by sample overloading for even minor contaminants to appear, confirmed high purity of the final product which predominantly consisted of F(ab')_2_ fragments since mostly peptides from the IgG heavy chain (constant region) and the light chain (variable region) were identified. In the most abundant protein spots on 2D gel, unique peptides from the IgM heavy chain were detected. The rest of the discrete protein spots contained only traces of transthyretin and inter-alpha-trypsin inhibitor (Fig. 3C, Additional file 1).

### Protective efficacy of F(ab')_2_ preparation

Neutralization potencies of HHP and F(ab')_2_-based preparation, together with specific activities of their IgGs or F(ab')_2_ fragments, are summarized in Table 2. According to the lethal toxicity neutralization assay in mice, no loss of the specific activity of the active drug in the final product with respect to that in starting material occurred. Purification factor of 3.9-fold, calculated as a ratio of specific activities of F(ab')_2_ preparation and HHP, was achieved (Table 2).

**Table 2.** *In vivo* neutralization potencies of HHP and F(ab')_2_ with specific activities. Purification factors obtained through manufacturing procedure are indicated. Results are expressed as means from *n* independently performed experiments +/- standard deviation.

		Plasma	Ultrapure F(ab')_2_
*R* ^a^	[LD_50_ mL^-1^]	44.0 ± 15.9 (*n* = 7)	94.1 ± 5.9 (*n* = 2)
*γ*(IgG) / (Fab')_2_^b^	[mg mL^-1^]	27.3 ± 2.3 (*n* = 9)	29.3 ± 0.2 (43.7 ± 0.3)^*^ (*n* = 3)
Specific activity of active drug^c^	[LD_50_ mg^-1^]	1.6 ± 0.6	2.2 ± 0.1
*γ*(protein)^d^	[mg mL^-1^]	58.2 ± 4.4 (*n* = 25)	30.1 ± 0.2 (*n* = 3)
Specific activity of sample^e^	[LD_50_ mg^-1^]	0.8 ± 0.3	3.1 ± 0.2
Purification factor^f^	[× ]	1.0	3.9

^a^ Lethal toxicity neutralization potency. ^b^IgG or F(ab')_2_ mass concentration. ^c^Specific activity of active drug in the sample calculated as ratio of *R* to *γ*(IgG) or *γ*(F(ab')_2_), respectively. ^d^Total protein mass concentration. ^e^Specific activity of the sample calculated as ratio of *R* to *γ*(protein). ^f^Purification factor calculated as ratio of specific activities of F(ab')_2_ product and HHP.

*Assumption of molecular weight reduction of the active drug due to Fc removal was considered

## Discussion

An effective strategy for antivenom preparation from equine plasma raised against *V. ammodytes ammodytes* venom is described. A highly stratified methodology was employed in which the elimination of the Fc part of IgGs occurred simultaneously with other plasma proteins, followed by further contaminant removal from the target compound, F(ab')_2_, by diafiltration and flow-through chromatography. Only three consecutive processing steps are sufficient to accomplish high yield, precisely quantified throughout the manufacturing procedure, and fulfilling the regulatory demands. In each phase the target compound was intentionally kept in solution to reduce the possibility of any conformational and/or structural change. The approach might be of great importance in large-scale manufacturing due to the ease of discarding precipitated unwanted proteins.

Initially, heat-treated plasma was exposed to the concurrent action of caprylic acid (2%, *V*/*V*) and pepsin (enzyme:IgG = 1:75, *w*/*w*) at pH 3.2 for 1.5 h. According to SDS-PAGE analysis, a temperature, set at 37 °C, proved unsupportive for precipitation of unwanted cleavage products (Fig. 1A). Furthermore, unexpectedly low total protein yield was obtained after diafiltration on a 50 kDa membrane, indicating that IgGs were probably over-digested. The single-reagent format has been conceptualized by Al-Abdulla et al. [17] who employed it for preparation of antivenom from ovine serum. Under conditions similar to ours, with the exception of a slightly higher pH (3.5) and longer incubation (4 h), they succeeded in obtaining F(ab')_2_-based product, reporting 94% purity following subsequent downstream processing steps, while a yield of around 58% could be calculated from provided data. Evidently, since two systems with their own specificities, including different immunoglobulin profiles, were involved, process terms established on ovine-originating starting material were not simply transferrable to equine plasma whose manipulation required an individualized approach. Therefore, in our work every step has been optimized *de novo* until finely tuned conditions for production of equine antivenom were achieved.

Lowering the temperature solved F(ab')_2_ contamination and loss issues (Fig. 1B), while use of pepsin at a higher concentration significantly improved yield (Figs. 1C and 1D). Under optimal conditions (temperature of 21 °C, enzyme: IgG = 1:30, *w*/*w*) only a 2 h incubation duration was sufficient to obtain crude F(ab')_2_ (Figs. 2B and 3A) with yield exceeding 70% (Table 1). It is noteworthy to mention the importance of the caprylic acid-mediated precipitation step in the overall purification process. Its implementation not only contributes to virus safety [20], but also, as we demonstrated, enables effective F(ab')_2_ preparation with rational pepsin amount even at a lower temperature, which opens up space for simplification of the production equipment and reduction of cost. For monitoring of the process efficiency and purity profiling, ELISA with sample-specific correction of results [32] for IgG quantification in plasma was preferentially chosen over other quantification methods such as SEC and densitometry, where overlapping of peaks/bands due to poor resolution or influence of protein type on surface area and intensity of developed color, respectively, is highly probable, leading to inaccurate result interpretation [35, 36, 37].

Enhancement of purity approaching 90% was achieved through diafiltration-mediated removal of small molecular fragments of most likely Fc, albumin and other plasma protein digestion by-products (Table 1). Surprisingly, SEC analysis revealed absence of aggregates from F(ab')_2_-enriched product, either crude (Fig. 2B) or pure (Fig. 2C). In our "first precipitation then cleavage" concept diafiltration as an intermediate step between caprylic acid fractionation and pepsin digestion was shown to be crucial for obtaining the final product of satisfactory composition [28]. Specifically, only removal of precipitating agent from IgG substrate prior to addition of enzyme ensured obtaining of F(ab')_2_ sample almost completely free of aggregates. In the refinement protocol presented herein, IgGs or F(ab')_2_ fragments remain protected from unwanted phenomena during their exposure to conditions involving mutual presence of caprylic acid and pepsin. Absence of aggregation might be associated with lower incubation temperature. 

Diafiltration was only partially effective for pepsin elimination, as already reported by others [26], and presented in our own research [28]. Therefore, a third purification step was introduced in which the equine plasma-derived F(ab')_2_ preparation was additionally polished by anion-exchange chromatography at pH 5.0 in the flow-through mode that enables efficient binding of residual acidic impurities, including the remaining pepsin, and unhindered passing of active drug [28]. The purity of 97% (Table 1) was somewhat poorer in comparison to that achieved by our originally developed downstream processing strategy, but is nevertheless within the range of methods published for some other F(ab')_2_ products which were prepared by various methodologies from hyperimmune plasma or serum on both the laboratory and manufacturing scale [16, 17, 27, 36, 38].

In order to get a deeper insight into contaminations of the final preparation, 2D gel electrophoresis and MS/MS analysis were performed (Fig. 3C, Additional file 1). This approach can also be considered as a further step for quality insurance. The great majority of protein spots have been attributed to IgG fragments. Some low-abundance protein spots, especially those of 15 kDa or less, were not successfully identified, either because of lack of material or insufficiently generated peptides.

An overall yield of around 74% (Table 1) was comparable to the process recovery obtained by our alternative scheme [28]. It appeared also as good as or even better than yields associated with enhanced-pepsin digestion (66-70%) and single-reagent protocols (56-60%) for refinement of ovine serum [17], assuming, as stated, that IgGs at concentrations between 30 and 32 g L^-1^ are commonly found in immunized sheep [21]. Although scarce, reports of efficiencies of downstream processing strategies for production of F(ab')_2_ antitoxins from pepsin-digested horse plasma are also in the literature, all being lower than the efficiency of the procedure herein described. When as additional steps either caprylic acid fractionation followed by ion-exchange chromatography [16] or combination of ion-exchange and affinity chromatography were employed [38], antibody activity recoveries of 65 and 41% were accomplished, respectively. Morais and Massaldi [39] reported that, under quite similar pH/time conditions, purified IgGs were cleaved to F(ab')_2_ fragments with 65% recovery of antigen-binding activity, measured by competitive ELISA stated to be in close correlation with ED_50_ assay.

Specific activity of F(ab')_2_ fragments at first appeared higher in comparison to that of the whole IgG molecules in starting plasma, although the difference is a consequence of protein content reduction in the final product due to Fc part removal. When the decrease in molecular weight of F(ab')_2_ fragments in relation to IgGs was considered, active drugs in the final product (F(ab')_2_) and in the respective plasma pool (IgG) were of comparable specific activities, proving that the Fc fraction is not relevant for the blocking of venom toxins. This finding supports our previous research [28] and agrees with the *in vivo* neutralization potency data provided by Segura et al. [36]. The fully preserved protective efficacy of F(ab')_2_ fragments, comparable to that of IgGs in plasma, implies that manufacturing process conditions did not affect significantly tertiary structure of the active drug nor provoked either loss of neutralizing IgG subclasses or redistribution of the venom-specific antibody content. 

## Conclusion

Fractionation of the venom-specific equine plasma employing compressed mode was independently repeated several times on two plasma pools of slightly different protective efficacy and by two analysts. Although appearing slightly inferior in comparison to our standard procedure with respect to purity, it resulted in an aggregate- and pepsin-free active drug with overall yield that was as good as or better than others so far reported. In addition to good physicochemical profile and high recovery of the product, the performance simplicity together with the short production cycle time in the laboratory setting were demonstrated. The suitability for large-scale antivenom manufacturing looks promising, but still needs to be addressed. 

## Supplementary material

The following online material is available for this article:

Additional file 1. (A) Two-dimensional gel electrophoresis (2DE) of F(ab')_2_-based final product (same sample as in Fig. 3C) with annotations of protein spots subjected to MS/MS analysis. (B) List of proteins identified in the final F(ab')_2_ sample. Proteins are denoted by the same numbers as in (A). Other protein spots remained unidentified.

## References

[B1] World Health Organization (2019). Snakebite envenoming: a strategy for prevention and control.

[B2] Gutierrez JM, Burnouf T, Harrison RA, Calvete JJ, Kuch U, Warrell DA (2014). A multicomponent strategy to improve the availability of
antivenom for treating snakebite envenoming. Bull World Health Organ.

[B3] Smith DC, Reddi KR, Laing G, Theakston RDG, Landon J (1992). An affinity purified ovine antivenom for the treatment of Vipera
berus envenoming. Toxicon.

[B4] Rawat S, Laing G, Smith DC, Theakston D, Landon J (1994). A new antivenom to treat Eastern coral snake (Micrurus fulvius
fulvius) envenoming. Toxicon.

[B5] Laing GD, Lee L, Smith DC, Landon J, Theakston RDG (1995). Experimental assessment of a new, low-cost antivenom for
treatment of carpet viper (Echis ocellatus) envenoming. Toxicon.

[B6] Rodrigues-Silva R, Martins MS, Magalhaes A, Santoro MM (1997). Purification and stability studies of immunoglobulins from
Lachesis muta muta antivenom. Toxicon.

[B7] Guidlolin RG, Marcelino RM, Gondo HH, Morais JF, Ferreira RA, Silva CL (2010). Polyvalent horse F(ab')2 snake antivenom: Development of process
to produce polyvalent horse F(ab')2 antibodies anti-african snake
venom. Afr J Biotechnol.

[B8] Rojas G, Jimenez JM, Gutierrez JM (1994). Caprylic acid fractionation of hyperimmune horse plasma:
description of a simple procedure for antivenom production. Toxicon.

[B9] Otero R, Gutierrez JM, Rojas G, Nunez V, Díaz A, Miranda E (1999). A randomized blinded clinical trial of two antivenoms, prepared
by caprylic acid or ammonium sulphate fractionation of IgG, in Bothrops and
Porthidium snake bites in Colombia: correlation between safety and
biochemical characteristics of antivenoms. Toxicon.

[B10] Steinbuch M, Audran R (1969). The isolation of IgG from mammalian sera with the aid of caprylic
acid. Arch Biochem Biophys.

[B11] Gutierrez JM, Rojas E, Quesada L, Leon GM, Núñez J, Laing GD (2005). Pan-African polyspecific antivenom produced by caprylic acid
purification of horse IgG: an alternative to the antivenom crisis in
Africa. Trans R Soc Trop Med Hyg.

[B12] Abubakar SB, Abubakar IS, Habib AG, Nasidi A, Durfa N, Yusuf PO (2010). Preclinical and preliminary dose-finding and safety studies to
identify candidate antivenoms for treatment of envenoming by saw-scaled or
carpet vipers (Echis ocellatus) in northern Nigeria. Toxicon.

[B13] Abubakar IS, Abubakar SB, Habib AG, Nasidi A, Durfa N, Yusuf PO (2010). Randomised controlled double-blind non-inferiority trial of two
antivenoms for saw-scaled or carpet viper (Echis ocellatus) envenoming in
Nigeria. PLoS Negl Trop Dis.

[B14] Vargas M, Segura A, Herrera M, Villalta M, Estrada R, Cerdas M (2011). Preclinical evaluation of caprylic acid-fractionated IgG
antivenom for the treatment of Taipan (Oxyuranus scutellatus) envenoming in
Papua New Guinea. PLoS Negl Trop Dis.

[B15] Al-Abdulla I, Casewell NR, Landon J (2013). Long-term physicochemical and immunological stability of a liquid
formulated intact ovine immunoglobulin-based antivenom. Toxicon.

[B16] Raweerith R, Ratanabanangkoon K (2003). Fractionation of equine antivenom using caprylic acid
precipitation in combination with cationic ion-exchange
chromatography. J Immunol Methods.

[B17] Al-Abdulla I, Casewell NR, Landon J (2014). Single-reagent one-step procedures for the purification of ovine
IgG, F(ab')2 and Fab antivenoms by caprylic acid. J Immun Methods.

[B18] Leon G, Monge M, Rojas E, Lomonte B, Gutierrez JM (2001). Comparison between IgG and F(ab')2 polyvalent antivnoms:
neutralization of systemic effect induced by Bothrops asper venom in mice,
extravasation to muscle tissue and potential for induction of adverse
reaction. Toxicon.

[B19] Garcia M, Monge M, Leon G, Lizano S, Segura E, Solano G (2002). Effect of preservatives of IgG aggregation, complement-activating
effect and hypotensive activity of horse polyvalent antivenoms used in
snakebite envenomation. Biologicals.

[B20] World Health Organization (2017). Expert Committee on Biological Standardization, Annex 5. Guidelines for
the production, control and regulation of snake antivenom
immunoglobulins.

[B21] Jones RGA, Landon J (2003). A Protocol for 'enhanced pepsin digestion': A step by step method
for obtaining pure antibody fragments in high yield from
serum. J Immunol Methods.

[B22] Hawgood BJ (2001). Poul Agerholm Christensen MD (1912-1991): antivenom production at
the South African Institute for Medical Research. Toxicon.

[B23] Gutierrez JM, Leon G, Burnouf T (2011). Antivenoms for the treatment of snakebite envenomings: the road
ahead. Biologicals.

[B24] León G, Vargas M, Segura Á, Herrera M, Villalta M, Sánchez A (2018). Current technology for the industrial manufacture of snake
antivenoms. Toxicon.

[B25] Gutierrez JM, Leon G, Lomonte B (2003). Pharmacokinetic-pharmacodynamic relationships of immunoglobulin
therapy for envenomation. Clin Pharmacokinet.

[B26] Jones RGA, Landon J (2002). Enhanced pepsin digestion: a novel process for purifying antibody
F(ab')2 fragments in high yield from serum. J Immunol Methods.

[B27] Kittipongwarakarn S, Hawe A, Tantipolphan R, Limsuwun K, Khomvilai S, Puttipipatkhachorn S (2011). New method to produce equine antirabies immunoglobulin F(ab')2
fragments from crude plasma in high quality and yield. Eur J Pharm Biopharm.

[B28] Kurtović T, Lang Balija M, Brgles M, Sviben D, Tunkic M, Cajner H (2019). Refinement strategy for antivenom preparation of high yield and
quality. PLoS Negl Trop Dis.

[B29] Eriksson L, Johansson E, Kettaneh-Wold N, Wikström C, Wold S (2000). Design of Experiments, Principles and
Applications. Umetrics AB (Stockholm).

[B30] Yüksel KU, Gracy RW (1985). The quantitation of proteins in silver stained polyacrylamide
gels. Electrophoresis.

[B31] Ehresmann B, Imbault P, Weil JH (1973). Spectrophotometric determination of protein concentration in cell
extracts containing tRNA's and rRNA's. Anal Biochem.

[B32] Halassy B, Kurtović T, Lang Balija M, Brgles M, Tunjic M, Sviben D (2019). Concept of sample-specific correction of immunoassay results for
precise and accurate IgG quantification in horse plasma. J Pharm Biomed Anal.

[B33] Kurtović T, Leonardi A, Lang Balija M, Brgles M, Habjanec L, Krizaj I (2012). The standard mouse assay of anti-venom quality does not measure
antibodies neutralising the haemorrhagic activity of Vipera ammodytes
venom. Toxicon.

[B34] European Pharmacopoeia (2016). Viper venom antiserum, European.

[B35] Hong P, Koza S, Bouvier ES (2012). Size-exclusion chromatography for the analysis of protein
biotherapeutics and their aggregates. J Liq Chromatogr Relat Technol.

[B36] Segura A, Herrera M, Villalta M, Vargas M, Gutiérrez JM, León G (2012). Assessment of snake antivenom purity by comparing physicochemical
and immunochemical methods. Biologicals.

[B37] Smejkal GB (2004). The Coomassie chronicles: past, present and future perspectives
in polyacrylamide gel staining. Expert Rev Proteomics.

[B38] Fernandes A, Kaundinya JO, Daftary G, Saxena L, Banerjee S, Pattnaik P (2008). Chromatographic purification of equine immunoglobulin G F(ab')2
from plasma. J Chromatogr B Analyt Technol Biomed Life Sci.

[B39] Morais V, Massaldi H (2005). Effect of pepsin digestion on the antivenom activity of equine
immunoglobulins. Toxicon.

