# Erratum: Streamlined downstream process for efficient and sustainable F(ab')_2_ antivenom preparation

**DOI:** 10.1590/1678-9199-JVATITD-2020-0025er

**Published:** 2020-10-09

In the article entitled “Streamlined downstream process for efficient and sustainable F(ab')_2_ antivenom preparation”, DOI: 10.1590/2358-2936e20200025, published in *Journal of Venomous Animals and Toxins including Tropical Diseases*, 2020, 26,

Page 1, in the title, where it was written:

“Streamlined downstream process for efficient and sustainable (Fab’)_2_ antivenom preparation”

Must be read:

“Streamlined downstream process for efficient and sustainable F(ab’)_2_ antivenom preparation”

And in page 8, Table 2, first column, where it was written:

“γ(IgG) / (Fab')_2_”

Must be read:

“γ(IgG) / F(ab')_2_”

The correction does not affect the discussion or conclusions of the original article.

