# Supplementary material for: Streamlined downstream process for efficient and sustainable (Fab')2 antivenom preparation
Source: J Venom Anim Toxins Incl Trop Dis. 2020 Jul 27;26:e20200025. doi: 10.1590/1678-9199-jvatitd-2020-0025 (PMC7384442; doi:10.1590/1678-9199-jvatitd-2020-0025)
Supplement: Additional file 1. [file 1678-9199-jvatitd-26-e20200025-s1.pdf]

J Venom Anim Toxins incl Trop Dis
https://doi.org/10.1590/1678-9199-JVATITD-2020-0025

Supplementary Material to "Streamlined downstream process for efficient and sustainable (Fab')$_2$ antivenom preparation"

Additional file 1. (A) Two-dimensional gel electrophoresis (2DE) of F(ab')$_2$-based final product (same sample as in Figure 3C) with annotations of protein spots subjected to MS/MS analysis. **(B)** List of proteins identified in the final F(ab')$_2$ sample. Proteins are denoted by the same numbers as in (A). Other protein spots remained unidentified.

(A)

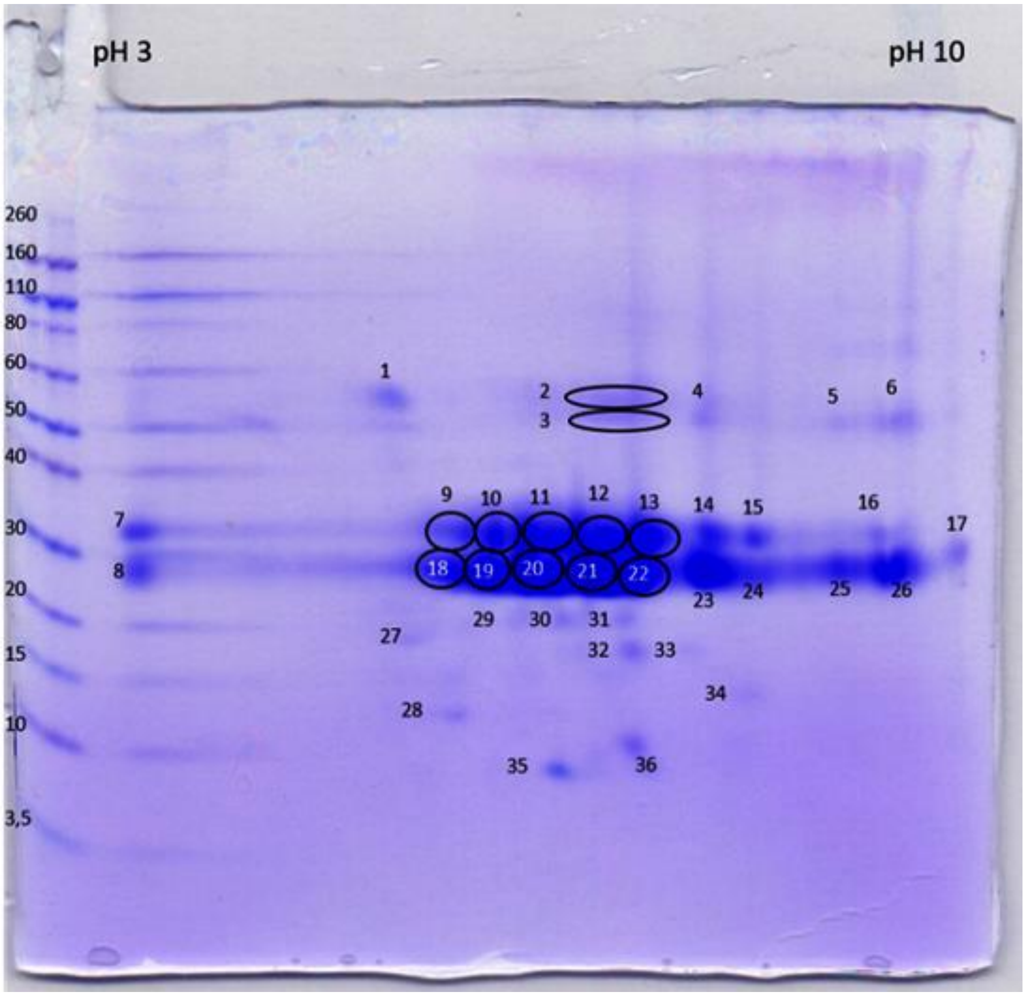

(B)

	Spot	Sample	Protein name	Protein ID	Protein score	Protein coverage	Significance	Peptide sequence
Identified high confidence	1	1_1424.72	Predicted: transthyretin	XP_014700846.1	40	-	only significant homology	AADETWELFASGK
		1_1663.85	transthyretin precursor	NP_001267607.1	40	-	only significant homology	TSEFGELHGLTTDEK
	5	5_1701.88	immunoglobulin lambda light chain V-J region, partial	ADK09711.1	77	6	yes	YAASSYLSLTPSQWK
		5_1701.88	Predicted: Low quality protein: immunoglobulin lambda-like polypeptide 5	XP_019832973.1	73	6	yes	YAASSYLSLTNSXWK
		5_1763.87	immunoglobulin gamma 6 heavy chain constant region, partial	CAC86341.1	54	-	only significant homology	SQTYICNVAHPASSTK
	7	7_2524.23	immunoglobulin heavy chain V-D-J region, partial	ADK09335.1	157	12	yes	SQVYLTLNSLTGEDTAVYYCAR
		7_2524.23	immunoglobulin mu heavy chain, partial	ARU82532.1	138	16	yes	SQVYLTLNSLTGEDTAVYYCVK
		7_2524.23	immunoglobulin mu heavy chain, partial	ARU82493.1	68	15	yes	DQFSLQLSSVTAEDTAVYYCAR
		7_2524.23	immunoglobulin mu heavy chain, partial	ARU82650.1	65	15	yes	SQVYLTLISLTGEDTAVYYCAR
		7_2852.37	immunoglobulin G heavy chain, partial	AAG01011.1	34	-	only significant homology	VPVGCLVSNYFPEPVTVSWNCDALK
	10	10_2524.24	immunoglobulin heavy chain V-D-J region, partial	ADK09335.1	75	12	yes	SQVYLTLNSLTGEDTAVYYCAR
		10_2524.24	immunoglobulin mu heavy chain, partial	ARU82532.1	60	16	yes	SQVYLTLNSLTGEDTAVYYCVK
		10_2820.39	immunoglobulin gamma 5 heavy chain constant region, partial	CAC86340.1	35	-	only significant homology	GDIHTFPLDLSNSAHHSLSSMMAVPR
		10_2851.39	immunoglobulin mu heavy chain G, partial	ARU82977.1	123	15	yes	VPVGCLVSNYFPEPVTVSWNCDALK
		10_2851.39	immunoglobulin G heavy chain, partial	AAG01011.1	123	5	yes	VPVGCLVSNYFPEPVTVSWNCDALK
	11	11_2524.21	immunoglobulin heavy chain, partial	AGJ50463.1	95	17	yes	SQVYLTLNSLTGEDTAVYVCAR
		11_2524.21	immunoglobulin mu heavy chain, partial	ARU82532.1	78	16	yes	SQVYLTLNSLTGEDTAVYYCVK
		11_2851.37	immunoglobulin G heavy chain, partial	AAG01011.1	128	5	yes	VPVGCLVSNYFPEPVTVSWNCDALK
		11_2851.37	immunoglobulin mu heavy chain G, partial	ARU82977.1	128	15	yes	VPVGCLVSNYFPEPVTVSWNCDALK
	13	13_2524.24	immunoglobulin heavy chain V-D-J region, partial	ADK09335.1	122	12	yes	SQVYLTLNSLTGEDTAVYYCAR
		13_2524.24	immunoglobulin mu heavy chain, partial	ARU82532.1	103	16	yes	SQVYLTLNSLTGEDTAVYYCVK
		13_2851.39	immunoglobulin G heavy chain, partial	AAG01011.1	179	5	yes	VPVGCLVSNYFPEPVTVSWNCDALK
		13_2851.39	immunoglobulin mu heavy chain G, partial	ARU82977.1	179	15	yes	VPVGCLVSNYFPEPVTVSWNCDALK
	14	14_1827.90	immunoglobulin G heavy chain, partial	AAG01011.1	64	3	yes	APDVFPLSICGNTPDPK
		14_1827.90	immunoglobulin mu heavy chain G, partial	ARU82977.1	64	10	yes	APDVFPLSICGNTPDPK
		14_2524.23	immunoglobulin heavy chain V-D-J region, partial	ADK09335.1	104	12	yes	SQVYLTLNSLTGEDTAVYYCAR
		14_2524.23	immunoglobulin mu heavy chain, partial	ARU82532.1	88	16	yes	SQVYLTLNSLTGEDTAVYYCVK
		14_2524.23	immunoglobulin mu heavy chain, partial	ARU82650.1	55	15	yes	SQVYLTLISLTGEDTAVYYCAR
	15	15_2524.24	immunoglobulin heavy chain V-D-J region, partial	ADK09335.1	112	12	yes	SQVYLTLNSLTGEDTAVYYCAR
		15_2524.24	immunoglobulin mu heavy chain, partial	ARU82532.1	96	16	yes	SQVYLTLNSLTGEDTAVYYCVK
		15_2851.38	immunoglobulin G heavy chain, partial	AAG01011.1	61	5	yes	VPVGCLVSNYFPEPVTVSWNCDALK
		15_2851.38	immunoglobulin mu heavy chain G, partial	ARU82977.1	61	15	yes	VPVGCLVSNYFPEPVTVSWNCDALK
	19	19_1701.86	immunoglobulin lambda light chain V-J region, partial	ADK09711.1	69	6	yes	YAASSYLSLTPSQWK
		19_1701.86	Predicted: Low quality protein: immunoglobulin lambda-like polypeptide 5	XP_019832973.1	65	6	yes	YAASSYLSLTNSXWK
		19_2851.35	immunoglobulin G heavy chain, partial	AAG01011.1	33	-	only significant homology	VPVGCLVSNYFPEPVTVSWNCDALK
	21	21_2221.16	immunoglobulin G light chain, partial	AAG01010.1	63	8	yes	ATVVCLISDFSPSGLEVIWK
		21_2221.16	immunoglobulin lambda light chain V-J region, partial	ADK09768.1	63	9	yes	ATVVCLISNFSPSGLEVIWK
		21_2851.36	immunoglobulin G heavy chain, partial	AAG01011.1	130	5	yes	VPVGCLVSNYFPEPVTVSWNCDALK
		21_2851.36	immunoglobulin mu heavy chain G, partial	ARU82977.1	130	15	yes	VPVGCLVSNYFPEPVTVSWNCDALK
Identified medium confidence	12	12_2851.34	immunoglobulin G heavy chain, partial	AAG01011.1	143	5	yes	VPVGCLVSNYFPEPVTVSWNCDALK
		12_2851.34	immunoglobulin mu heavy chain G, partial	ARU82977.1	143	15	yes	VPVGCLVSNYFPEPVTVSWNCDALK
	16	16_1701.88	Predicted: Low quality protein: immunoglobulin lambda-like polypeptide 5	XP_019832973.1	82	6	yes	YAASSYLSLTNSXWK
		16_1701.88	immunoglobulin lambda light chain V-J region, partial	ADK09711.1	79	6	yes	YAASSYLSLTPSQWK
	17	17_1701.84	immunoglobulin lambda light chain V-J region, partial	ADK09711.1	63	6	yes	YAASSYLSLTPSQWK
	20	20_2820.37	immunoglobulin mu heavy chain G, partial	ARU82985.1	84	14	yes	GDIHTFPLDLSNSAHHSLSSMMAVPR
		20_2820.37	immunoglobulin G heavy chain, partial	AAG01011.1	84	6	yes	GDIHTFPLDLSNSAHHSLSSMMAVPR
		20_2820.37	immunoglobulin gamma 5 heavy chain constant region, partial	CAC86340.1	84	7	yes	GDIHTFPLDLSNSAHHSLSSMMAVPR
	22	22_2221.17	immunoglobulin lambda light chain V-J region, partial	ADK09768.1	89	9	yes	ATVVCLISNFSPSGLEVIWK
		22_2221.17	immunoglobulin G light chain, partial	AAG01010.1	89	8	yes	ATVVCLISDFSPSGLEVIWK
	23	23_2221.16	immunoglobulin G light chain, partial	AAG01010.1	91	8	yes	ATVVCLISDFSPSGLEVIWK
		23_2221.16	immunoglobulin lambda light chain V-J region, partial	ADK09768.1	91	9	yes	ATVVCLISNFSPSGLEVIWK
	25	25_1701.84	immunoglobulin lambda light chain V-J region, partial	ADK09711.1	71	6	yes	YAASSYLSLTPSQWK
		25_1701.84	Predicted: Low quality protein: immunoglobulin lambda-like polypeptide 5	XP_019832973.1	68	6	yes	YAASSYLSLTNSXWK
	26	26_1701.85	Predicted: Low quality protein: immunoglobulin lambda-like polypeptide 5	XP_019832973.1	57	6	yes	YAASSYLTNSXWK
		26_1701.85	immunoglobulin lambda light chain V-J region, partial	ADK09711.1	57	6	yes	YAASSYLSLTPSQWK
	27	27_1655.99	Inter-alpha-trypsin inhibitor	P04365.2	58	12	yes	TVAACNLPIVQGPCR
		27_1655.99	protein AMBP	XP_001488384.1	58	4	yes	TVAACNLPIVQGPCR
		27_1655.99	protein AMBP	XP_014704695.1	58	4	yes	TVAACNLPIVQGPCR
	30	30_1763.67	immunoglobulin gamma 6 heavy chain constant region, partial	CAC86341.1	53	-	only significant homology	SQTYICNVAHPASSTK
	31	31_1763.80	immunoglobulin gamma 7 heavy chain, partial	AAS18414.1	67	4	yes	SQTYICNVAHPASSTK
	32	32_1763.65	immunoglobulin gamma 7 heavy chain, partial	AAS18414.1	85	4	yes	SQTYICNVAHPASSTK
	34	34_1764.03	immunoglobulin gamma 7 heavy chain, partial	AAS18414.1	87	4	yes	SQTYICNVAHPASSTK
